# Anterior right hepatic artery pseudoaneurysm secondary to biliary tract instrumentation treated by angioembolization

**DOI:** 10.1093/jscr/rjaf603

**Published:** 2025-08-06

**Authors:** Carmen Judith Roca Vásquez, Carlos Alberto Córdova Velázquez, Gabrielle Nandayapa Pérez, Ratziel Alberto Lezama Molina, Jorge Alberto Roldán García, Oscar Chapa Azuela

**Affiliations:** Universidad Nacional Autónoma de México, México City, México; General Surgery Department, Hepatopancreatobiliary Surgery, Hospital General de México “Dr. Eduardo Liceaga”, México City, México; Universidad Nacional Autónoma de México, México City, México; General Surgery Department, Hepatopancreatobiliary Surgery, Hospital General de México “Dr. Eduardo Liceaga”, México City, México; Universidad Nacional Autónoma de México, México City, México; General Surgery Department, Hepatopancreatobiliary Surgery, Hospital General de México “Dr. Eduardo Liceaga”, México City, México; Universidad Nacional Autónoma de México, México City, México; General Surgery Department, Hepatopancreatobiliary Surgery, Hospital General de México “Dr. Eduardo Liceaga”, México City, México; Universidad Nacional Autónoma de México, México City, México; General Surgery Department, Hepatopancreatobiliary Surgery, Hospital General de México “Dr. Eduardo Liceaga”, México City, México; Universidad Nacional Autónoma de México, México City, México; General Surgery Department, Hepatopancreatobiliary Surgery, Hospital General de México “Dr. Eduardo Liceaga”, México City, México

**Keywords:** hepatic artery, pseudoaneurysm, hepaticojejunostomy, angioembolization

## Abstract

Hepatic artery pseudoaneurysm is a rare complication following biliary tract instrumentation. Clinical presentation may range from asymptomatic course to catastrophic hemorrhage with high morbidity and mortality. We report a case of anterior right hepatic artery pseudoaneurysm secondary to surgical instrumentation. A 71-year-old woman underwent hepaticojejunostomy due to a Strasberg E5 bile duct injury. In the postoperative period, she presented with gastrointestinal bleeding and hemodynamic instability. Angio-CT revealed a saccular lesion in a branch of the right hepatic artery (Segment V). Angiography confirmed the diagnosis, and selective embolization of the pseudoaneurysm was successfully performed using microcoils. This case illustrates a rare complication following biliary tract instrumentation and discusses diagnostic and therapeutic options for hepatic artery pseudoaneurysms.

## Introduction

A hepatic artery pseudoaneurysm (HAP) is a rare complication following biliary tract instrumentation. HAP can occur due to surgical trauma, percutaneous interventions, liver transplantation, biliary fistula, and have also been reported after cholecystectomy. Its incidence is underestimated as many cases are asymptomatic and go unreported. An incidence between 0.06% and 0.6% has been reported in laparoscopic procedures involving the cystic artery [1]. Treatment includes angioembolization; if unsuccessful, surgical intervention may be indicated [2]. This case is presented following the SCARE criteria [3].

## Case report

In April 2024, a 71-year-old woman with no relevant medical history presented with acute cholecystitis requiring emergency laparoscopic cholecystectomy, complicated by a Strasberg E5 bile duct injury. Initially, four percutaneous catheters were placed: two in collections (short-term) and two in the biliary tract—one in the posterior right hepatic duct (PRHD) and another in the anterior right hepatic duct (ARHD), which also drained the left segments. Preoperative laboratories were within normal parameters. Angio-CT showed no vascular damage or anatomical variants. Percutaneous transcatheter cholangiography demonstrated drainage of the anterior right and left bile ducts through one catheter and the posterior right duct through another. Cholangioresonance imaging showed separation of the right hepatic duct, with the ARHD and left hepatic duct (LHD) not communicating with the common hepatic duct or common bile duct.

**Figure 1:**
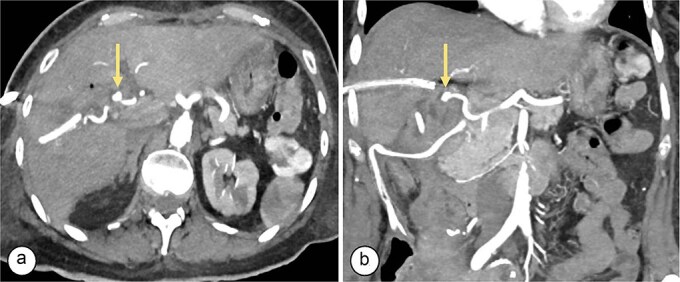
Angiotomography. (a) Axial slice showing a saccular image (arrow) with an aneurysmal appearance, 8 × 5 mm in size, originating from the right hepatic artery (Segment V), with no evidence of active bleeding. (b) Coronal slice showing a pseudoaneurysm (arrow) in the branch of the right hepatic artery (Segment V).

**Figure 2:**
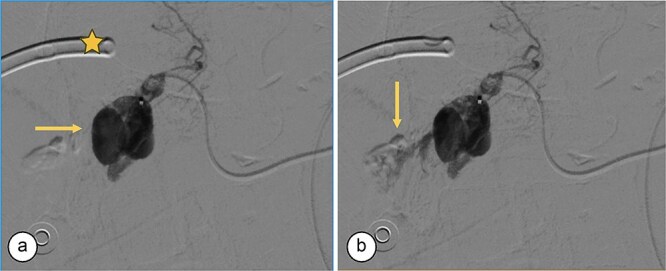
Angiography. (a) Saccular image consistent with a hepatic artery pseudoaneurysm (Segment V). (b) Pre-embolization image of the right hepatic artery showing contrast medium leakage (arrow) from a branch (Segment V) of the right hepatic artery. Right percutaneous catheter (star).

**Figure 3:**
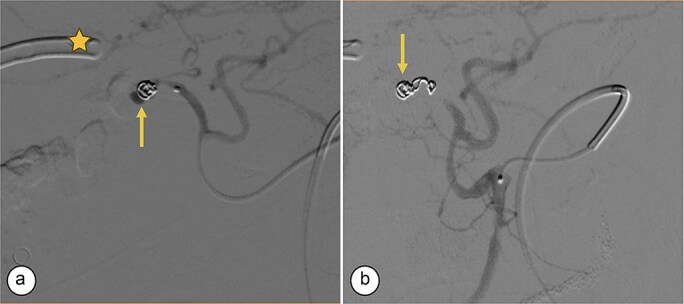
Transcatheter arterial embolization of the right hepatic artery. (a) Selective transcatheter arterial embolization (TAE) of the branch of the right hepatic artery (Segment V) (arrow). (b) Post-embolization image of the pseudoaneurysm in the branch (Segment V) of the right hepatic artery with microcoils (arrow), showing no contrast medium leakage. Right percutaneous catheter (star).

**Figure 4:**
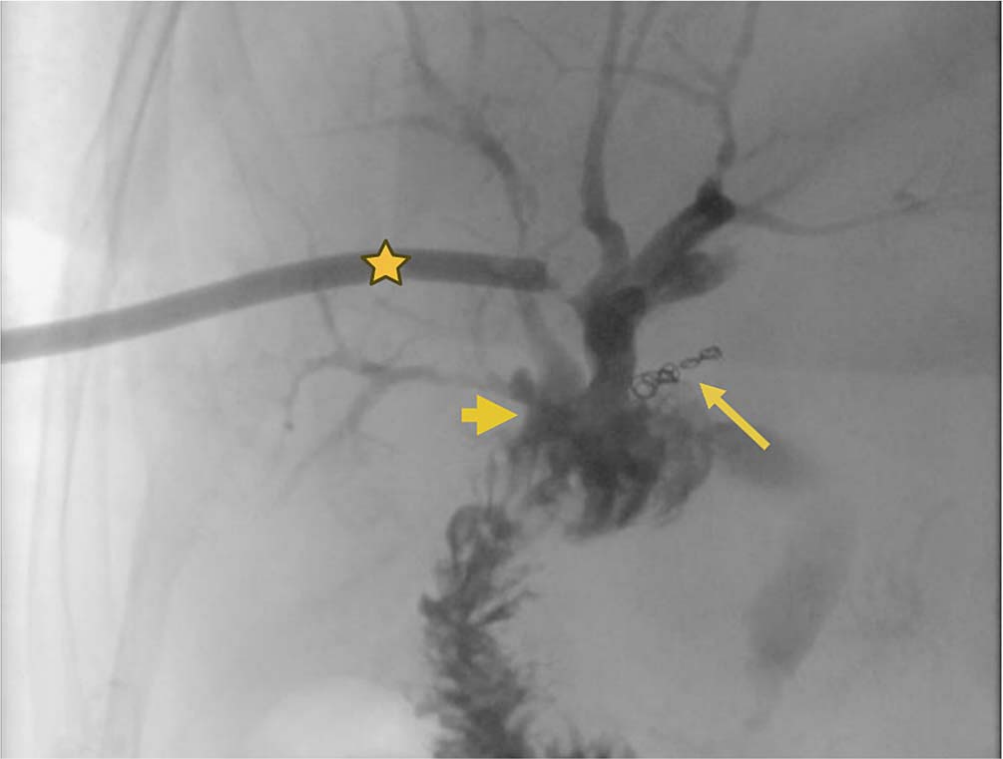
Supranastomotic percutaneous transcatheter cholangiography. Hepaticojejunostomy (arrowhead), microcoils (arrow), and percutaneous catheter (star).

Surgical reconstruction revealed an excluded PRHD and ARHD–LHD continuity with obstruction 10 mm from the confluence. The PRHD catheter was found outside the biliary tract, so a transhepatic Nelaton catheter was placed from inside to out. A neoconfluence of PRHD with ARHD and LHD was created with a 30 mm hepaticojejunostomy. A transanastomotic Nelaton catheter and a supranastomotic ARHD catheter were left in place. Estimated blood loss: 300 cc. In the immediate postoperative period, the patient developed melena and bleeding from the Penrose drain. Cholangiography via the right percutaneous catheter showed no contrast leakage. Angio-CT revealed an 8 × 5 mm saccular lesion in the anterior right hepatic artery (Segment V), consistent with a pseudoaneurysm (Figs 1 and 2). Selective embolization with three microcoils (2 × 3 × 2.3 mm) was successfully performed (Fig. 3). The patient had a favorable recovery, with no further bleeding, and was discharged in stable condition for outpatient follow-up. Control imaging of the ARHD catheter confirmed adequate visualization of all hepatic ducts, allowing catheter removal (Fig. 4).

## Discussion

HAP is a rare complication of biliary instrumentation that may lead to hemorrhage and increased morbidity and mortality. Visceral aneurysms are rare, with a prevalence of 1%. The most commonly affected visceral arteries include the splenic, hepatic, gastroduodenal, pancreaticoduodenal, superior mesenteric, and left gastric arteries [4]. HAPs rank second among visceral aneurysms, comprising 20% of cases. Most HAPs are extrahepatic (75%–80%), primarily affecting the common hepatic artery (63%). Branch pseudoaneurysms are less common: 23% involve the right hepatic artery, 5% the left, and 4% are bilateral [5]. Delayed presentation may result from unnoticed surgical injury or a local inflammatory process. Risk factors include mechanical trauma during surgery (dissection or thermal injury), bile leakage, or arterial irritation from infected intra-abdominal collections causing arterial wall erosion [6, 7]. HAPs have been reported following liver transplantation, pancreatic surgery, and biliodigestive anastomoses [8]. Bile duct injuries increase the risk of vascular damage to the right hepatic artery, with pseudoaneurysm prevalence in this context ranging from 2.6% to 4.5% [9]. HAPs often manifest as hemobilia—the most common sign (64%)—followed by hematemesis (30%), hematochezia (14%), abdominal pain (20%), or may be asymptomatic [10]. Quincke’s triad—jaundice, biliary colic, and gastrointestinal bleeding—has also been described [11]. Our patient presented with GI bleeding and hemodynamic instability.

Diagnostic modalities to localize GI bleeding include upper endoscopy [12]. In this case, angio-CT revealed a saccular lesion in an anterior branch of the right hepatic artery (Segment V), and angiography confirmed the diagnosis.

HAP is a medical emergency requiring immediate intervention. Rupture risk ranges from 21% to 80%, with a 50% mortality rate. Open surgical repair carries greater morbidity compared to endovascular techniques. Therefore, endovascular approaches should be prioritized in anatomically suitable patients [13]. Selective hepatic arterial embolization is the first-line treatment for symptomatic HAP using microcoils, gelatin sponge pledges, or inflatable balloons. Advantages include rapid intervention, selectivity, minimal invasiveness, and repeatability [14].

Surgery is not first-line and is reserved for embolization failure or if the bleeding source cannot be identified. Surgical options include arterial ligation and resection. Other alternatives include image-guided percutaneous thrombin injection [14].

HAP is a rare but serious complication of biliary tract instrumentation. Any patient presenting with GI bleeding and hemodynamic deterioration should be evaluated for potential pseudoaneurysm.

In conclusion, patients with HAP after biliary instrumentation may be asymptomatic or present with GI bleeding and hemodynamic compromise, significantly increasing morbidity and mortality. A high index of suspicion is essential for timely diagnosis and prompt therapeutic intervention. Treatment options include angioembolization, percutaneous thrombin injection, or surgery.
